# From TLS architecture to functional immune niches: division of labor among CAFs, HEVs, and antigen-presenting cells in cancer immunotherapy

**DOI:** 10.3389/fimmu.2026.1958533

**Published:** 2026-09-28

**Authors:** Li Xiao, Hongbo Qiu, Honglin Luo

**Affiliations:** 1 Department of Laboratory Medicine, Zigong First People’s Hospital, Zigong, Sichuan, China; 2 Department of Oncology, Zigong First People’s Hospital, Zigong, Sichuan, China

**Keywords:** cancer immunotherapy, cancer-associated fibroblast, fibroblastic reticular cell, high endothelial venule, lymphotoxin beta receptor, tertiary lymphoid structure

## Abstract

Tertiary lymphoid structures (TLSs) are associated with antitumor immunity and have prompted efforts to induce lymphoid organization within tumors. The central interpretive problem is whether the resulting tissue changes contribute to treatment benefit or accompany effects mediated by other cells. Building on established TLS biology, this Review compares the cellular targets, functional measurements, and antitumor outcomes of stromal, endothelial, and antigen-presenting-cell interventions, with particular attention to cancer-associated fibroblasts (CAFs). Across experimental systems, defined CAF states support chemokine production and lymphocyte recruitment, whereas vascular entry and antigen-dependent T-cell responses are demonstrated through distinct interventions. The synthesis distinguishes a stromal contribution to immune organization from evidence that this contribution is required for tumor control, preserving the model and anatomical setting in which each relationship was tested. Mechanistic support comes mainly from murine and engineered systems, while human studies chiefly establish spatial relationships and clinical associations. For therapies that remodel TLS-associated niches, this comparison identifies the induced functions whose contribution to treatment benefit needs to be tested before tissue changes are used to guide clinical treatment.

## 1 Introduction

Efforts to improve cancer immunotherapy increasingly include inducing or remodeling tertiary lymphoid structures (TLSs), where lymphocytes and antigen-presenting cells can assemble into local immune niches. Across several cancers, TLSs are associated with favorable outcomes and responses to immune checkpoint inhibition, providing a rationale for this approach (1). The experimental question, however, is whether an induced structure contributes to the antitumor response or accompanies other treatment effects. In murine tumors, lymphotoxin beta receptor (LTβR) agonism enhanced lymphocyte entry through peripheral node addressin (PNAd)-positive vessels even in settings without fully developed TLSs (2). In a separate engineered model, lymph-node-derived stromal cells supported T-cell responses in peritumoral structures whose sparse B cells did not form follicles (3). These findings broaden the relevant tissue configurations without making follicular organization unimportant. They shift attention to the function being sustained in a particular niche and the cells responsible for it.

Cancer-associated fibroblasts (CAFs) are especially relevant because stromal interventions can alter the recruitment and organization of lymphocytes. Human spatial studies identify fibroblastic reticular cell-like (FRC-like) CAF populations and related fibroblast states in lymphocyte-rich regions, although their markers and classification methods differ among studies (4–6). Functional experiments resolve a more specific contribution. In pancreatic cancer, reticular and myofibroblastic CAF phenotypes defined before treatment differ in their response to LTβR stimulation. Inhibiting transforming growth factor-beta (TGFβ)/ALK5 signaling relieves suppression of chemokine production and B-cell recruitment, while combined TGFβR1 inhibition and LTβR agonism improves tumor control in resistant models (7). High endothelial venule (HEV) studies address a different function, the passage of lymphocytes from blood into tissue (8, 9). Interventions involving conventional type 1 dendritic cells (cDC1) and vascular remodeling provide evidence of antigen-dependent responses or T-cell-dependent tumor control in separate systems (10, 11). These findings concern different cellular actions and, in some studies, different anatomical compartments. Their interpretation depends on matching the manipulated population to the measured outcome while accounting for exchange between tumors and draining lymphoid tissues.

Existing syntheses already place these functions within a broader account of TLS biology. Teillaud and colleagues integrated TLS developmental states with effector and regulatory T- and B-cell functions, clinical associations, and therapeutic manipulation (1). Other reviews organized the evidence around maturity-based classification and induction strategies, immune-cell composition and structural heterogeneity, or experimental models for therapeutic exploitation, with particular attention to lung cancer (12–14). Stromal mechanisms have also received detailed treatment. Radandish and colleagues reviewed fibroblast and endothelial contributions to TLS formation and immune regulation (15), while Tang and colleagues linked CAF plasticity and TLS organization to immune exclusion, antigen presentation, and treatment response (16). Importantly, Tang and colleagues also considered alternative causal relationships between CAFs and TLSs and recognized that architecture alone does not ensure productive antitumor immunity. Reviews of vascular normalization-TLS coupling and the multicellular actions of LTβR further explain how vascular state, signaling context, and treatment influence these interactions (17, 18). Functional interpretation and causal uncertainty are therefore part of the existing knowledge base. The narrower question pursued here is how strongly each reported immune effect can be assigned to the cellular component that was actually altered.

This Review compares what happens after a defined cellular component is altered: which recruitment or activation function changes, and whether the antitumor effect depends on that change. Its contribution is a critical comparison of functional attribution across TLS-associated niches, building on established TLS biology rather than proposing another developmental model. The evidence points to a context-dependent stromal contribution: a recruitment-supporting CAF state can facilitate immune organization, while endothelial and antigen-presenting-cell functions have their own experimental determinants. Findings from different models are kept distinct so that similar tissue appearances do not imply a shared treatment mechanism. Human studies establish the clinical associations and anatomical settings in which these mechanisms are worth testing. The intended use is to distinguish competing explanations for an intervention’s immune effects and guide focused mechanistic studies; clinical treatment selection requires separate validation.

Literature scope and evidence appraisal. This focused narrative review was assembled from a local reference library and initial searches of PubMed/MEDLINE and Web of Science Core Collection conducted on 28 June 2026. During revision, targeted supplementary searches and citation verification were conducted through 5 September 2026 to address the current focus on CAF/FRC-like states, HEV-associated entry, cDC1 and other antigen-presenting cells, TCF1-positive CD8 T cells, lymphatic and tumor-draining-node context, and cancer immunotherapy. The search combined concept blocks for tertiary lymphoid structures/TLS, cancer-associated fibroblasts or FRC-like fibroblasts, HEV/PNAd, cDC1 or other antigen-presenting cells, TCF1-positive CD8 T cells, lymphatic or tumor-draining-node context, and cancer immunotherapy. We included peer-reviewed human tumour studies, translational studies, and mechanism-defining cancer models that directly addressed these processes. Non-tumour models were used only when they clarified a mechanism. Preprints and abstract-only records were not used for central claims, tables, or figures. Studies were prioritized when they linked a cellular or spatial feature to a functional readout or perturbation; spatial co-occurrence was retained as descriptive evidence, whereas mechanistic and clinical interpretations required corresponding intervention, temporal, comparative, or outcome data. The synthesis is descriptive and purposive, not a systematic review, and does not claim exhaustive retrieval or formal risk-of-bias grading.

Evidence is discussed using four evidence settings that are not treated as interchangeable. Descriptive evidence includes spatial co-occurrence, morphology and cell-state profiles. Functional evidence requires a direct readout such as migration, antigen presentation, proliferation, cytokine production or cytotoxicity. Mechanistic or causal evidence requires a targeted perturbation of the candidate component together with a corresponding change in the readout; temporal ordering, a matched comparison, rescue or an orthogonal perturbation can strengthen that inference but does not replace the perturbation itself. Clinical evidence refers to observations in patient material or treatment cohorts and may be descriptive or functional; clinical setting alone does not establish causality or treatment mediation. Each subsection identifies both the evidence setting and the inference supported by the study before drawing a conclusion.

## 2 Evidence for CAF participation in TLS-associated immune organization

Throughout the manuscript, “mature TLS” is reserved for structures whose source study documents the relevant combination of B- and T-cell compartmentalization, follicular dendritic-cell organization and, when present, a germinal centre. “TLS-like structure” and “lymphoid aggregate” are retained when those criteria are incomplete or not reported. Maturity describes architectural organization; it does not establish lymphocyte trafficking, antigen presentation, T-cell reactivation, or tumor control. Conversely, an incomplete structure may still support a measured immune function. A “candidate HEV” denotes a vessel with a reported HEV-associated phenotype or morphology; PNAd/MECA-79 staining, endothelial shape, vessel continuity and demonstrable blood-to-tissue trafficking are recorded as separate observations rather than collapsed into one label.

### 2.1 Spatial CAF states identify candidate organizing niches

Human spatial studies consistently place selected fibroblast states within lymphocyte-rich regions. In colorectal cancer liver metastases, CCL19-producing fibroblasts lie close to CCR7-positive B and T cells (19). Head and neck squamous cell carcinomas contain a CCL19/CCL21-enriched FRC-like population associated with B cells, non-regulatory CD4 T cells, and TLS regions (4). These observations converge on a stromal neighborhood containing both lymphocyte-positioning signals and the cells that can respond to them. Cross-cancer single-cell spatial analyses extend this finding by identifying a recurrent lymphoid-neighborhood CAF state with chemokine and antigen-presentation-associated programs (5). The common feature is the location of a defined fibroblast state within an immune-cell neighborhood, rather than uniform expression of one marker across tumors. This provides an anatomical basis for investigating a stromal contribution to lymphoid organization.

Cell identity determines how that spatial association is interpreted. The FRC-like population described in head and neck cancer combines CCL19 and CCL21 with VCAM1, RBP5, and SPIB, whereas the colorectal liver-metastasis study identifies CCL19-producing cells within a fibroblast compartment (4, 19). The cross-cancer classification is instead derived from cellular neighborhoods and molecular programs (5). These approaches identify related features at different analytical scales; their labels do not establish a single interchangeable CAF population. Similarly, a chemokine-rich tissue region does not identify the producing cell unless the signal is assigned to a defined cellular compartment. Cell-resolved annotation therefore distinguishes a candidate fibroblast contribution from a mixed stromal and immune signal. It also preserves the difference between expression of an antigen-presentation program and a measured T-cell response.

The anatomical unit being sampled adds a second distinction. Three-dimensional reconstruction of a colorectal tumor showed that lymphoid regions appearing separate in two-dimensional sections could belong to a larger connected network, with graded differences in cellular composition across that network (20). In such a specimen, several apparent aggregates need not represent independent niches, and a fibroblast’s relationship to one section may omit its wider tissue context. This detailed reconstruction illustrates a sampling problem rather than a universal topology for cancer TLSs. Spatial studies are thus most informative when cell identity, neighborhood composition, and the extent of the sampled structure are considered together. They localize a plausible organizing population; whether that population actively recruits or supports lymphocytes is addressed by perturbation experiments.

### 2.2 CAF perturbations support stromal recruitment but do not establish the entire immune pathway

Pancreatic cancer experiments connect a pre-existing fibroblast state to a measured recruitment response. Before LTβR stimulation, permissive tumor models were enriched in ICAM1-positive, VCAM1-positive reticular CAFs, whereas resistant models contained more myofibroblastic CAFs with higher αSMA and activated SMAD2 (7). The distinction therefore preceded the response to treatment. In fibroblast cultures, TGFβ-induced myofibroblastic programming suppressed lymphotoxin/TNF-associated chemokine expression and migration of B cells and T-cell subsets toward fibroblasts. TGFβR1 inhibition relieved suppression of chemokine expression and B-cell migration in TNFα/LTβR-stimulated fibroblasts. In TLS-resistant tumors, combined TGFβR1 inhibition and LTβR agonism shifted CAF phenotypes and improved tumor control; conditional Alk5 deletion in the Pdgfra-expressing fibroblast compartment further linked the effect to stromal signaling (7). These complementary experiments connect baseline phenotype, fibroblast-derived recruitment signals, and an intervention that changes the phenotype. They provide more direct evidence of CAF participation than an increase in fibroblasts and lymphocytes observed after treatment alone.

Other stromal experiments resolve different aspects of organization. In colorectal liver-metastasis models, CCL19-induced B-cell aggregation was sensitive to CCR7 blockade, while combining CCL19 with CXCL13 produced stronger lymphoid organization than CCL19 alone (19). This identifies a chemokine-dependent recruitment mechanism whose architectural outcome depends on additional signals. An engineered transplantation study addresses a different question: a lymph-node-derived FRC-like stromal line generated ectopic lymphoid structures before adjacent tumor implantation (3). The structures contained T-cell clusters, whereas the scarce B cells did not form follicles. Tumor-lysate dendritic-cell vaccination elicited functional responses from T cells recovered from these structures. This demonstrates organizing capacity in an implanted stromal compartment, not the developmental origin or necessity of endogenous tumor CAFs. Taken together, chemokine blockade and stromal transplantation distinguish dependence on a recruitment signal from the capacity of an introduced stromal population to assemble an immune-cell structure.

T-cell dependence of tumor control was tested separately from stromal recruitment. In the pancreatic cancer study, combined CD4/CD8 T-cell depletion abolished the benefit of LTβR agonism plus TGFβR1 inhibition. Under the same combined treatment, CXCL13 blockade reduced B-cell infiltration without impairing efficacy. In a separate TLS-permissive model, anti-CD20-mediated B-cell depletion did not reverse the benefit of LTβR agonism alone (7). These results establish T-cell dependence of the combined regimen and show that the tested B-cell-directed interventions did not abolish efficacy in their respective settings. They do not isolate a CD8-only mechanism or selectively test the requirement for a particular lymphoid structure. PNAd-blockade experiments in separate tumor and engineered HEV-transplantation models address lymphocyte entry from blood (2, 9), rather than CAF necessity. Distinguishing these interventions assigns the evidence to stromal recruitment, vascular entry, or immune effector activity as appropriate. **Table 1** compares these stromal, endothelial, APC, and lymphocyte contributions. Across the stromal studies, the clearest CAF contribution is to chemotaxis, adhesion, and early organization, with its extent depending on fibroblast state and tissue context.

**Table 1:** Process-level evidence across stromal, vascular and antigen-presenting-cell compartments in TLS-associated niches.

Biological process	Cells and signals	Principal findings	Evidence context	References
Induction of lymphoid stromal programs	Fibroblasts; LTβR, TNF and TGFβ signaling	Lymphotoxin and TNF induce chemokine and adhesion programs. TGFβ-conditioned myofibroblastic states show reduced responsiveness.	Human fibroblast cultures; neonatal dermal fibroblasts; murine pancreatic cancer and melanoma. Ligand format and baseline state influence the response.	(4, 7, 21, 22)
CCR7-dependent lymphocyte recruitment	CCL19/CCL21-producing stroma; CCR7-positive lymphocytes (CAF-specific contribution where tested)	CCL19-positive fibroblast proximity identifies a candidate stromal source in human tumors; CAF-specific chemokine manipulation or migration assays support recruitment only where directly tested.	Human spatial associations; murine liver-metastasis and pancreatic-cancer experiments. CCR7 also contributes to vascular entry in melanoma models.	(4, 7, 19, 23)
B-cell aggregation and follicular organization	CXCL13-producing stroma, CD4 T cells and FDCs; CXCR5; CCR7 ligands (source-specific)	Chemokine combinations promote lymphoid aggregation in experimental systems. CXCL13 sources differ between early and follicle-containing TLSs, so aggregation is not assigned uniformly to CAFs.	Human source localization; murine melanoma, ovarian cancer and liver metastases. Chemokine-induced aggregates vary in structural completeness.	(19, 22, 24, 25)
Adhesion and intratissue migration	Reticular fibroblasts or other stromal cells; ICAM1, VCAM1 and collagen architecture (fibroblast-specific where tested)	Stimulated fibroblasts increase lymphocyte attachment in defined assays. Reticular networks and matrix alignment accompany tissue organization, but matrix-guided motility was measured outside a TLS system.	Fibroblast cocultures, melanoma tissue and three-dimensional collagen assays.	(7, 21, 22, 26)
HEV-like endothelial differentiation	Endothelial cells with stromal or immune partners; LTβR/TNFR pathways	Receptor stimulation or FRC–endothelial assembly induces specialized vascular phenotypes. This is an endothelial process and is not assigned to CAFs without a CAF-specific test.	Murine tumors and engineered human endothelial organoids. FAP-targeted delivery localizes stimulation but does not isolate CAF signaling.	(2, 9, 23, 27)
Lymphocyte entry from blood	Specialized endothelium; luminal PNAd and lymphocyte L-selectin	PNAd-dependent trafficking supports lymphocyte entry. Blocking this interaction reduces recruitment in tumor and organoid models.	Short-term homing and MECA-79 blockade. Effects differ by lymphocyte subset and anatomical site; vessel staining and entry are separate readouts.	(2, 9, 23)
APC-associated CD8 T-cell responses	DCs, including cDC1; antigen presentation and FLT3L	DC interventions enhance antigen-presentation or CD8 effector readouts. Vascular remodeling can enrich APC–TCF1-positive CD8 neighborhoods as a parallel context, not as a CAF-mediated step.	DC assays, murine lung-cancer vaccination and glioma imaging. The activation site is not resolved by APC proximity or TCF1 expression.	(2, 10, 11)
CD4-mediated support of CD8 responses	MHC-II-positive CAFs, CD4 T cells and Tpex CD8 T cells	CAF-conditioned CD4 cells enhance CD8 responses in gastric-cancer cocultures. A distinct CD4 state supports Tpex cells in an antigen-dependent mouse model.	Human spatial observations, serial cocultures and adoptive transfer. These are distinct helper interactions, rather than one established CAF–CD4–Tpex pathway.	(6, 28)
B-cell support and antibody production	B cells, plasma cells and neighboring stroma	Stromal state alters B-cell adhesion or proliferation in defined systems. Plasma-cell localization and tumour-binding IgG are B-lineage outputs in other contexts, not CAF-mediated antibody production.	Ovarian stromal cocultures, cross-cancer spatial data and human IgG assays. Antibody production is a B-lineage output, distinct from CAF recruitment.	(5, 19, 29)

The table is a process-level summary of multiple cellular contributors; its rows are not claims that CAFs mediate every process. TLS-associated niches include organized TLSs and less complete lymphoid or perivascular aggregates. Experimental systems and anatomical locations differ among the cited studies. APC, antigen-presenting cell; CAF, cancer-associated fibroblast; cDC1, conventional type 1 dendritic cell; DC, dendritic cell; FDC, follicular dendritic cell; HEV, high endothelial venule; PNAd, peripheral node addressin; TCF1, T-cell factor 1; TLS, tertiary lymphoid structure.

## 3 CAF states that support TLS formation

### 3.1 FRC-like CAFs are not a fixed cell type

The fibroblast labels used here are operational and source-specific. FRC-like CAF refers to a tumor-associated fibroblast population annotated from a reported marker or transcriptional program resembling lymph-node fibroblastic reticular cells; the label is not assigned because the population responds to LTβR stimulation. Immunofibroblast describes an immune-organizing fibroblast population in its source tissue: in Sjögren’s syndrome, it identified a PDPN-positive, FAP-positive network with distinct chemokine-producing and lymphocyte-survival-supporting subsets (30). Lymphoid-like CAF is retained as a descriptive tumor-stromal annotation based on the original study’s lymphoid-associated molecular or spatial features. These terms can overlap with FRC-like programs without defining the same cell population. CCL19-positive or CCL21-positive fibroblasts identify a reported output or marker, whereas FAP-positive and perivascular fibroblast labels identify a marker-defined or anatomical population. Lymph-node stromal cells used in engineered models are not interchangeable with endogenous tumor CAFs. The human spatial studies discussed above illustrate related chemokine and neighborhood features obtained using different classification approaches (4, 5, 19). Transcriptional similarity can identify a recurrent state without establishing a shared developmental origin, a fixed CAF subtype, comparable state duration, or an equivalent response to LTβR, TNF, TGFβ, or other signals. Across studies, the comparable unit is therefore the measured feature in its tissue and experimental context—such as chemokine production, adhesion, matrix organization, antigen presentation, or vascular proximity—not the label itself.

FRC-like states also vary with tissue location. As noted in Section 2.1, three-dimensional reconstruction of colorectal cancer showed that apparently separate TLS-like regions can belong to one connected network with graded immune composition (20). In high-grade serous ovarian cancer, TLS features also depend on anatomical site and adjacent stroma. Regions rich in PDPN-positive CAFs do not always coincide with high CCL19, CCL21, and CCR7 expression (29, 31). An FRC-like CAF is not an isolated marker combination, but a state embedded within a particular tissue location and immune composition.

Treatment, anatomy, and analytical resolution further influence the observed state. Treatment-associated regions in bladder cancer and left- versus right-sided colorectal cancers contain different chemokine networks. Similar CAF programs can be recombined according to treatment context and tumor location (32, 33). In bulk transcriptomic data, NOVA1 covaries with several cellular components and can localize a relevant region without identifying the cellular source (34). CCL21-positive CAFs in penile squamous cell carcinoma and post-neoadjuvant changes in inflammatory and myofibroblastic CAFs in pancreatic cancer likewise show association with TLS formation or treatment remodeling, but not the temporal order of state transitions (35, 36). Cross-cohort comparisons are further distorted when studies count ungraded lymphoid aggregates under different TLS definitions (37, 38). A more robust approach first compares shared features, including CCL19/CCL21 expression, adhesion programs, and lymphocyte neighborhoods. Antigen-presentation, matrix, vascular, and inflammatory contexts can then be recorded separately (5, 20).

### 3.2 TGFβ and local inflammation determine CAF responsiveness

The pre-existing CAF state governs its response to lymphotoxin. Pancreatic ductal adenocarcinoma models that formed lymphoid aggregates after LTβR agonism were enriched before treatment for ICAM1-positive, VCAM1-positive reticular CAFs, whereas resistant models contained predominantly myofibroblastic CAFs. TGFβ-driven myofibroblastic programming suppresses Cxcl13, Ccl19, and VCAM1 and reduces support for cellular migration. Inhibition of TGFβR1 or fibroblast-specific Alk5 deletion restores LTβR responsiveness and T-cell-dependent tumor control (7). This study provides direct evidence that one signal can produce divergent outcomes in different CAF states. Its implication is not that CAFs form permanently “favorable” and “unfavorable” classes. Rather, myofibroblastic contraction, extracellular-matrix production, and lymphoid chemotaxis compete for cellular resources and transcriptional output. Both states may coexist within one tumor, with their relative abundance and spatial distribution shaping the local outcome. Human studies should report not only the frequency of a CAF population, but also its location within the tumor core, invasive margin, or periphery of TLSs, and whether treatment produces a concordant state transition.

Human tissue and other models provide directionally consistent but less direct support. Combined stimulation induces part of an FRC-like program in human oral fibroblasts, while CAFs with different FAP states are associated with divergent patterns of immune organization in melanoma (4, 22). FAP is inversely associated with TLSs in esophageal cancer, and a trajectory of CXCL12-positive CAFs accompanies colorectal cancer progression. These observations do not establish FAP or CXCL12 as cross-cancer classifiers of immune-supportive and immune-suppressive CAFs (39, 40). TGFβ should likewise not be treated as a uniformly suppressive switch. Its effect depends on the responding cell, the ligand dose and form, the tissue state, and the time window: in the pancreatic model it restrains a reticular CAF program, whereas in other stromal or immune contexts TGFβ can participate in tissue repair, matrix remodeling, or regulatory-cell differentiation (7, 41). The available evidence therefore supports a variable balance between reticular and myofibroblastic programs, with TGFβ acting as one context-dependent regulator rather than a binary patient-stratification marker.

Signals from neighboring immune cells, tumor cells, and nerves further shape CAF behavior. Cancer tissue containing lymphoid stroma can simultaneously harbor TGFβ-associated contacts between dendritic cells and regulatory T cells. TLSs in soft-tissue sarcoma may likewise be embedded in a macrophage-maintained suppressive environment (41, 42). Tumor-cell FAK activity in ovarian cancer limits lymphocyte entry and the CXCL13 program, whereas sensory denervation promotes early TLS-like structures and CD8 T-cell-dependent tumor control (43, 44). In a tobacco-exposure model, tumor-cell AhR signaling increases CCL21, showing that a critical chemokine need not originate from CAFs (45). These studies explain why similar FRC-like CAFs may accompany different outcomes. CAF state matters, but it remains subject to several concurrent stimulatory and inhibitory signals.

Because immune cells can alter CAFs in return, initiating signals must be distinguished from later feedback. Time-resolved perturbation in melanoma separated CD8 T-cell initiation, B-cell expansion, and late LTβR-dependent amplification. The dominant driver can change as the tissue develops (22). IL-33-ILC2 interactions in pancreatic cancer, reciprocal communication between human antigen-presenting CAFs and macrophages, and other myeloid-stromal circuits further show that initiation, CAF maintenance, and immune effector activity can be distributed among different cells (6, 46–48). The central questions are consequently more informative than a longer list of FRC markers: which signals first alter CAFs, which cells maintain that state, and whether CAF-derived chemotactic and adhesive programs change cellular entry and local immunity.

## 4 CAF-mediated immune-cell recruitment and organization

### 4.1 LTβR and TNFR transmit inflammatory signals to CAFs

Once CAFs acquire an FRC-like state, they still require organizing signals from immune cells. Activation of an “LTβR pathway” is not sufficient information because ligand form, cellular source, and duration determine the response. In fibroblast cultures, soluble TNFα induces mainly transient adhesion-molecule expression. Membrane-bound LTα1β2 combined with TNFα produces stronger and more sustained expression of ICAM1, VCAM1, Cxcl13, Ccl19, and Ccl21 (21). Engineered nanovesicles induce antigen-presenting cells to supply LTα and TNFα, followed by vascular and lymphoid-tissue remodeling. LTβR blockade attenuates these changes and reduces treatment activity, implicating local ligand supply in tissue organization (49). Soluble LIGHT also increases intratumoral immune cells and delays tumor growth. However, LIGHT acts on stromal, endothelial, myeloid, and T cells, preventing attribution of the entire response to CAFs or TLSs (50). LTA-positive memory B cells outside melanoma aggregates identify B cells as another potential source of these signals (51).

The same ligand can still evoke different CAF responses. The pancreatic cancer study paired tumor-model comparisons with fibroblast cultures: TNFα plus LTβR agonism induced chemokine and adhesion programs in culture, whereas TGFβ pretreatment suppressed this induction. In resistant tumors, combining TGFβR1 inhibition with LTβR agonism restored early lymphocyte aggregation and T-cell-dependent tumor control (7). Combined stimulation also induces partial FRC-like features in human oral fibroblasts, indicating plasticity without proving that CAFs in patient tumors undergo the same transition (4). TGFβ-associated immunosuppression, macrophage-rich environments, and expansion of CXCL12-positive CAFs can also oppose an organizing signal (40–42). LTβR and TNFR provide initiating inputs, whereas the pre-existing CAF state and surrounding inhibitory signals determine whether those inputs produce recruitment and tissue reorganization.

### 4.2 Chemokines position distinct immune-cell populations

Expression of CCL19, CCL21, and CXCL13 directs different immune populations towards the same region. CCL19 and CCL21 primarily act on CCR7-positive T cells and dendritic cells, whereas CXCL13 attracts CXCR5-positive B cells. In colorectal cancer liver metastases, CCL19 alone increases lymphocyte aggregation. Combining CCL19 with CXCL13 more readily produces T-cell/B-cell compartmentalization and local antibody responses (19). In the Colon26 model, chemokines alone mainly generate B-cell aggregates. Distinct compartmentalization and HEV-associated vessels become clearer when lymphotoxin signaling is also present (2). Combined delivery of CXCL13 and CCL21 similarly increases TLS-like structures in melanoma and improves tumor control during anti-PD-L1 treatment (25). Multiple chemokine axes can increase encounters among immune-cell populations without ensuring mature TLS formation. Absolute chemokine abundance is not the only determinant. Cells migrate along spatial gradients, so the location of a signal on the vascular lumen, around vessels, or within deep stroma affects arrest and subsequent movement. Broad and uniform overexpression may instead erase directional information. Outcomes also depend on receptor expression on incoming cells, the capacity to cross basement membrane, and local retention signals. A complete analysis should measure ligand source, spatial gradient, receptor-bearing cells, and actual migration rather than compare transcript abundance alone.

CAFs are not the sole source of these chemokines. Activated CD4 T cells in early ovarian cancer TLSs produce CXCL13, as do CD21-positive cells or CD8 T cells in different regions of endometrial cancer (24, 52). Immune cells are the main source of CXCL10 in ectopic lymphoid tissues associated with prostate cancer. Tumor-cell FAK activity and GATA6-positive macrophages also shape the CXCL13 environment in ovarian cancer (43, 47, 53). Detecting CXCL13 or CCL21 establishes a local recruitment signal, but not its CAF origin. In human tissue, assigning chemokine sources remains difficult because secreted proteins can be detected away from their producing cells, while bulk expression measurements combine signals from multiple cell populations. Multiplex *in-situ* detection linked to cell-resolved annotation, spatial transcriptomics with validated cell markers, and cell-specific perturbation provide a more reliable route to source assignment than expression abundance alone.

After arrival, immune cells must be retained and organized. ICAM1, VCAM1, and their integrin partners support adhesion, while PDPN-positive reticular extensions and collagen fibers influence migration routes. Transplanted FRC-like cells create T-cell-dominant reticular structures beside tumors. Three-dimensional collagen studies further show that fiber alignment strongly affects cytotoxic T-cell migration (3, 26). Matrix organization has opposing effects. A loose, continuous reticular scaffold promotes movement and cellular encounters, whereas excessive crosslinking or unfavorable collagen alignment lengthens migration paths and creates exclusion. Adhesion also requires an appropriate strength. Insufficient adhesion prevents retention, while excessive adhesion may restrict subsequent antigen search. Chemokines bring cells into proximity; adhesion molecules and matrix networks determine whether they remain, interact, and form stable regions. Only when these processes connect with a vascular gateway and antigen presentation can local aggregation support a sustained immune response.

### 4.3 Tissue organization emerges through a multicellular relay

No single signal completes TLS-associated organization. In a non-malignant sialadenitis model, IL-13/IL-4R first induces adhesive immunofibroblasts, followed by IL-22/IL-22R-dependent network expansion. Early changes can occur without intact LTβR signaling, whereas later chemotactic programs and ordered structures depend more strongly on lymphotoxin circuits (30). Tumor models show related stage-specific effects. In B16-OVA tumors, effector lymphocytes induce PNAd through the LTα3-TNFR axis, while IFNγ primarily regulates CCL21. These signals separately control vascular adhesion and local chemotaxis (23). Engineered T-bet/IL-36γ dendritic cells first increase T-cell entry, after which PNAd-positive vessels emerge. IL-36R blockade attenuates later vascular changes and infiltration, whereas LTβR blockade during a defined window does not eliminate the early therapeutic effect (54). In human colorectal cancer, vascular IL-36γ correlates mainly with B-cell density inside established TLSs. It may amplify an existing structure rather than initiate one across tumors (55).

Other cells and tissue features can intervene at different stages. Sensory denervation promotes early TLS-like aggregates in melanoma. Vascular LIGHT can first improve perfusion and immune-cell entry, followed by immature lymphoid tissue (44, 56). These findings are compatible because initiation, expansion, cellular positioning, vascular remodeling, and long-term maintenance can be performed sequentially by different cells. Identifying the responsible step requires intervention before, during, and after structure formation, with serial measurements of CAF state, cell location, vascular trafficking, and local immune function. Once recruitment and positioning are established, the next question is whether circulating lymphocytes can actually enter these regions through tumor vessels.

## 5 HEV-mediated lymphocyte entry into tumors

### 5.1 PNAd positivity indicates a potential gateway, not proven trafficking

HEVs are commonly identified by PNAd or MECA-79 staining, but positive staining shows only a phenotype resembling lymph-node HEVs. A more defensible candidate designation combines the reported endothelial phenotype with supportive morphology, including plump or cuboidal endothelial cells, a continuous luminal lining and an anatomically plausible vessel. LTβR agonism induces PNAd-positive vessels across several murine tumors, while fully developed TLSs form in only some models (2). In the 4NQO mouse model of oral carcinogenesis, PNAd-positive vessels appeared in early lesions without B-cell aggregates meeting the study’s TLS definition; their trafficking function was not tested (57). After treatment with engineered T-bet dendritic cells, T-cell entry precedes the increase in PNAd-positive vessels. Cellular entry, vascular differentiation and TLS maturation therefore do not follow a fixed sequence (54). In human breast cancer, some TLS-associated vessels lack MECA-79 but express sialylated Lewis X, TSPAN7 or MEOX2. These features may identify specialized or HEV-like venules, but they do not justify naming an HEV without compatible morphology and evidence of blood-to-tissue trafficking (58).

Endothelial subtyping in human tumors further shows that similar HEV morphologies can represent different functional states. Interferon-responsive HEVs in nasopharyngeal cancer are associated with CXCR3-positive lymphocyte aggregation and immunotherapy response. An ID1-high endothelial state in lung cancer similarly accompanies durable response and post-treatment remodeling (8, 59). By contrast, mature HEVs expressing checkpoint ligands coexist with lower CD8 infiltration and adverse outcomes in another setting. Higher densities of MECA-79-positive vessels are associated with better survival in intrahepatic cholangiocarcinoma (60, 61). HEVs cannot be classified as favorable or unfavorable by a single marker. Distinguishing HEVs from bile ducts and conventional tumor vessels is especially important in tissues such as the liver. Evidence based only on CD34 and smooth-muscle-actin images described as “HEV-like vessels” warrants particular caution (62, 63).

### 5.2 Trafficking and blockade experiments directly test gateway function

Demonstrating that an HEV is a lymphocyte gateway requires short-term movement from blood into the tumor, with local proliferation and redistribution from adjacent tissue excluded. Transplanted HEV organoids derived from human pluripotent stem cells recruit B cells and both CD4 and CD8 T cells. MECA-79 blockade markedly reduces entry, providing direct evidence of PNAd-dependent transvascular migration (9). In B16-OVA tumors, short-term homing assays were combined with restricted peripheral recirculation and blockade of L-selectin, CCR7, LFA-1 and luminal PNAd. These experiments showed that naive CD8 T cells enter through lymph-node-like vessels and contribute to the subsequent antitumor response (23). Thus, a vessel label, a trafficking phenotype and a functional gateway should be reported as separate evidence layers.

These studies also demonstrate that HEV formation is not controlled by one pathway. In B16-OVA tumors, LTα3-TNFR signaling primarily regulates PNAd, whereas IFNγ has a larger effect on CCL21. In the engineered dendritic-cell model, IL-36R blockade reduces both PNAd-positive vessels and T-cell infiltration (23, 54). Bladder cancer provides an opposing example. An IGLL5-associated B-cell state suppresses LTβR signaling, disrupts HEVs, and destabilizes TLSs. Intervening in this axis restores organization and immune activity (64). LTβR, TNFR, IFNγ, and IL-36 can affect adhesion, chemotaxis, or vascular maintenance at different times and in different models. None should be presented as a universal HEV switch across cancers.

### 5.3 Effective HEVs depend on endothelial state

A functional gateway must capture, arrest, and transmit lymphocytes. Endothelial adhesion molecules mediate capture, luminal chemokines guide arrest and migration, and pericytes and basement membrane influence vascular stability. Cells must then be retained and activated after crossing the vessel. Requirements also differ among lymphocyte states. Naive and central memory T cells rely more strongly on L-selectin and CCR7, whereas activated effector T cells can use additional inflammatory adhesion and chemokine axes. The same HEV change may preferentially increase one T-cell population, a compositional shift that total CD8 counts cannot resolve. Greater vascular flux may also fail to improve efficacy when the incoming cells are predominantly tumor-irrelevant bystanders. Disruption at any step can produce PNAd-positive vessels with little trafficking, or successful entry without an effective response. Studies should report vascular phenotype, the state of incoming cells, directional migration, and the immune consequences of blockade rather than treating HEV density as a complete functional measure. Existing HEV reviews have standardized concepts related to PNAd expression, vascular maintenance, and remodeling. TLS or HEV thresholds derived within individual cohorts still require validation in independent patients (65, 66).

Associations between HEVs, TLSs, or endothelial states and clinical outcomes can nominate biomarkers but do not show that altered trafficking mediates therapeutic benefit. Small baseline analyses in oral cancer, exceptional responses in renal cancer with low TLS transcriptional signals, small trial-associated molecular subsets, and single-arm treatment cohorts lack formal treatment-interaction or mediation analyses (67–70). The next step is not simply a larger sample. Endothelial state, actual lymphocyte entry, and local immunity must be measured before and during treatment in the same patient. Even when an HEV transports lymphocytes, therapeutic benefit still depends on whether those cells re-encounter antigen and receive sufficient activating signals.

## 6 Local antigen presentation and T-cell reactivation

### 6.1 cDC1 provide a defined cross-presentation route

cDC1 connect antigen capture and cross-presentation with the local CD8 T-cell response, but the evidence needs to be separated by anatomical step. In several models, FLT3L-supported cDC1 increase in tumor-draining lymph nodes and subsequently appear in the tumor, where they are associated with stronger CD8 T-cell activation and durable memory (10). In the LTβR-agonist model, vascular and stromal remodeling coincided with increased activated dendritic cells and TCF1-positive, PD-1-positive CD8 T cells; antigen-presentation assays, restricted peripheral recirculation, CD8 depletion, and MECA-79 blockade provided distinct evidence for local presentation, effector-cell dependence, and vascular entry (2). These experiments support a route in which cDC1 can deliver a second antigenic stimulus after lymphocytes have entered, but they do not show that every TCF1-positive cell was reactivated inside the tumor or that cDC1 were the sole presenting population. The strongest interpretation comes from combining antigen-specific readouts with cDC1 perturbation, T-cell-receptor clonotype tracking, and measurements made before and after entry through the tumor vasculature.

Other cDC1-directed interventions illustrate why the local stimulus should not be equated with a single marker. Vascular-targeted LIGHT produces perivascular regions containing APCs and TCF1-like CD8 T cells in glioma, with tumor control dependent on CD8 T cells and the LIGHT receptor (11). FAP-targeted delivery of an LTβR agonist increases HEVs, deep intratumoral T cells, and TCF1-positive CD8 T cells while enhancing checkpoint therapy (27). These studies establish that vascular remodeling, antigen-presenting-cell abundance, and T-cell state can change together, but the intervention does not isolate the cDC1 contribution. TCF1 or PD-1 positivity therefore remains a state descriptor. Evidence for local reactivation is stronger when antigen-dependent APC–T-cell contact is accompanied by T-cell-receptor signaling, proliferation, recovery of effector function, or a clonal relationship to cells that subsequently mediate tumor control.

TCF1 positivity identifies a progenitor-like or memory-associated state, but it does not by itself demonstrate antigen-dependent reactivation. A local reactivation claim requires an antigen-linked functional readout, such as T-cell-receptor signaling, proliferation, cytokine production, cytotoxicity, or clonal continuity after the relevant APC or antigen is perturbed.

**Figure 1** organizes evidence from complementary models around two contrasting CAF states. When a TGFβ-associated myofibroblastic program predominates, CAFs are less able to translate lymphotoxin and TNF signals into chemotaxis, adhesion, and vascular coordination. Responsive CAFs can instead support immune-cell recruitment, which may coexist with HEV-mediated trafficking and contacts between cDC1 and CD8 T cells. These functions form a cross-model working framework, not a fixed temporal sequence. No cited study simultaneously validates CAF responsiveness, HEV trafficking, cDC1 contact, and tumor control.

**Figure 1:**
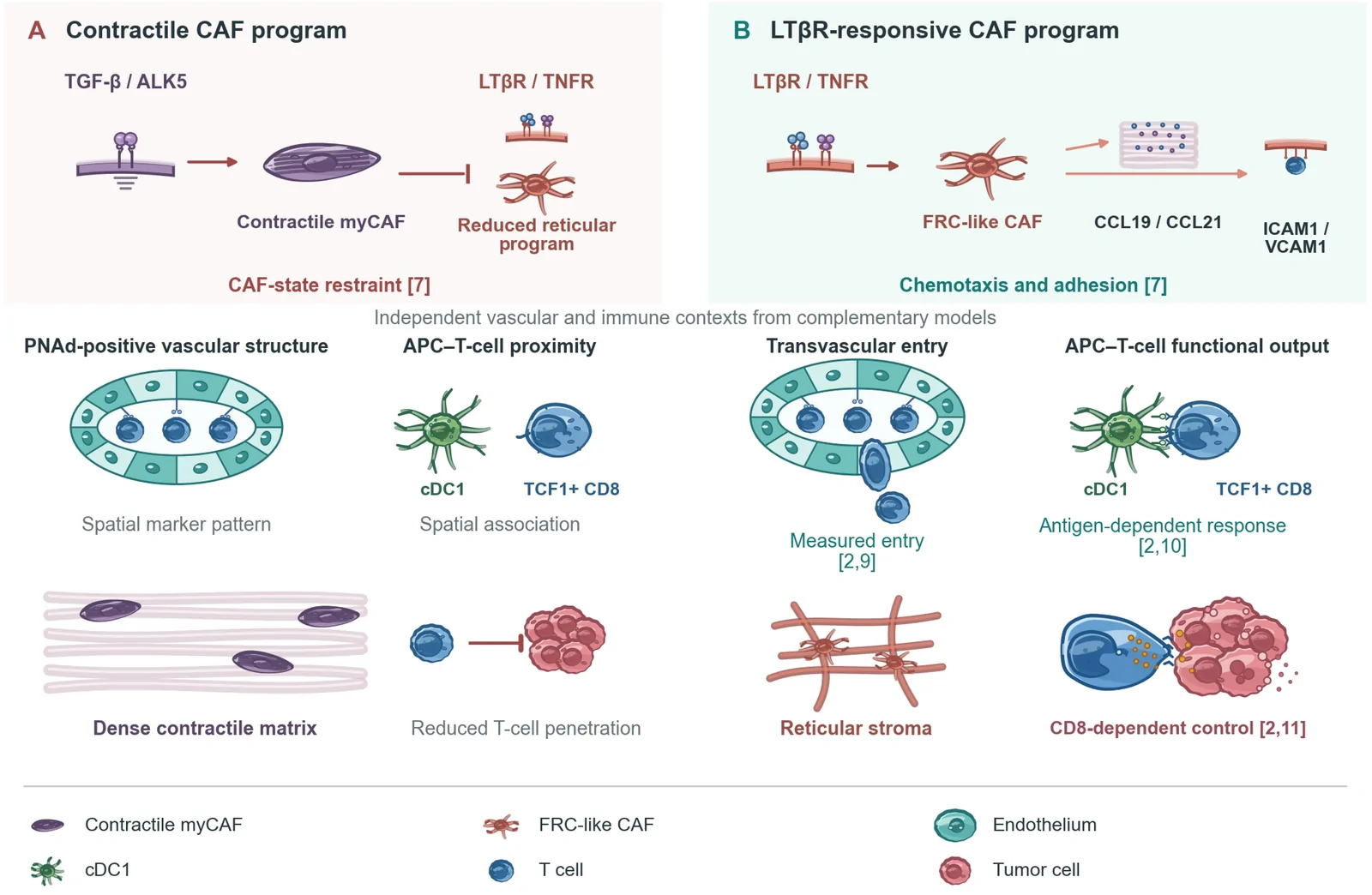
CAF-state responsiveness and distinct vascular and immune functions. The upper diagrams compare a TGF-beta/ALK5-associated contractile myCAF program **(A)** with an LTbetaR/TNFR-responsive CAF program **(B)** that displays reticular features. In pancreatic cancer models, myofibroblastic programming restrains chemokine and adhesion responses, whereas TGF-beta receptor inhibition or fibroblast-specific Alk5 deletion restores LTbetaR responsiveness (7). This comparison concerns CAF state and does not place the downstream observations in a single causal sequence. Arrowheads indicate local signaling or stromal output, and blunt-ended lines indicate restraint. In the lower-left structural context, the dense contractile matrix is shown with a restrained path toward the tumor, indicating reduced T-cell penetration rather than complete exclusion. The lower scenes are deliberately labeled by evidence type: PNAd-positive vascular structure and cell proximity are spatial observations, whereas transvascular entry, antigen-dependent APC-T-cell output, and CD8-dependent tumor control are independently measured functions (2, 9–11). The absence of arrows between these scenes is intentional because they synthesize complementary models rather than outputs of one CAF perturbation. Reference 7 supports T-cell-dependent control through combined CD4/CD8 depletion rather than isolated CD8 dependence. cDC1 illustrates one presenting route; reference 2 does not isolate cDC1 as the sole APC. TCF1 denotes cell state rather than demonstrated reactivation or obligatory effector differentiation. Vessels are shown in oblique section with a continuous endothelial lining and luminally anchored PNAd-bearing addressins. Reference 9 uses engineered transplantation to clarify trafficking, not to establish a mechanism in patients with cancer. FRC-like CAF denotes a source-defined reticular marker or transcriptional program and is not asserted to be lineage-equivalent to lymph-node FRCs. Cell identities are shown in the key. ALK5, activin receptor-like kinase 5; APC, antigen-presenting cell; CAF, cancer-associated fibroblast; cDC1, conventional type 1 dendritic cell; FRC, fibroblastic reticular cell; HEV, high endothelial venule; LTbetaR, lymphotoxin-beta receptor; myCAF, myofibroblastic CAF; PNAd, peripheral node addressin; TCF1, T-cell factor 1; TGF-beta, transforming growth factor-beta; TNFR, tumor necrosis factor receptor.

### 6.2 Non-cDC1 APCs contribute through context-dependent presentation and support

cDC1 are not the only cells that can shape antigen-dependent T-cell activity in a TLS-associated niche. Other conventional dendritic cells may provide antigen presentation or costimulation without demonstrating the same cross-presentation capacity as cDC1. Macrophages can maintain chemokine-rich and compartmentalized regions, and their depletion can disrupt B-cell/T-cell organization and increase tumor burden (71). B cells and CD4 T cells may supply antigen presentation, costimulation, or lymphotoxin-family signals that sustain local niches; in melanoma and other tumors, these interactions can support T-cell persistence without proving that B cells or CD4 T cells initiate the response (28, 51). Antigen-presenting CAFs have also been reported, but their ability to display antigen should not be treated as evidence that they replace professional APCs or drive CD8 T-cell reactivation in every tumor (6). Each population must therefore be assigned the function directly measured in the source study: antigen presentation, costimulation, chemokine maintenance, or structural support.

The anatomical route further separates these roles. FLT3L-secreting cDC1 can act across tumor-draining lymph nodes and the tumor, linking systemic T-cell replenishment with intratumoral stimulation (10). In brain metastases from non-small cell lung cancer, TCF1-positive CD4 T cells and microglia jointly shape TLS-like aggregates associated with treatment response, illustrating that a non-cDC1 myeloid or lymphoid compartment can organize a local niche in a tissue-specific manner (72). CXCL13-producing immune cells, including activated CD4 T cells and macrophages, can position T cells independently of CAF-derived chemokines (24, 47, 52, 73). These findings broaden the cellular sources of support, but they do not establish a common APC hierarchy. A cell that maintains a chemokine field or provides costimulation may be necessary for persistence while remaining dispensable for initial cross-presentation.

An incomplete lymphoid structure can consequently remain functional, but function cannot be inferred from architectural incompleteness alone. T-cell responses in engineered reticular structures and early tumor aggregates have been measured by IFNγ production, cytotoxicity, clonal continuity, or tumor control (3, 74, 75). Conversely, CXCL13-positive CD8 T cells or TCF1-positive cells can occur in regions with low antigen-presentation activity or high checkpoint expression, where proximity is compatible with suppression rather than productive activation (76, 77). The relevant comparison is not mature versus immature TLS. It is whether the local APC population presents the relevant antigen, provides the required costimulation, and is linked to a measurable T-cell response.

### 6.3 Local T-cell output requires more than APC proximity

A functional APC–T-cell niche should be evaluated as a sequence of linked observations rather than a spatial label. First, the presenting cell and the antigen source should be identified at cellular resolution. Second, contact or shared localization should be paired with a readout of T-cell-receptor engagement, proliferation, cytokine production, or cytotoxicity. Third, perturbing the candidate APC, its antigen-presentation machinery, or the relevant antigen should weaken the T-cell response; rescue or an orthogonal perturbation strengthens the inference. Restricted recirculation and clonal tracking can further indicate whether the response is sustained locally, although they do not by themselves identify the presenting cell (2). This sequence distinguishes cDC1-dependent cross-presentation from non-cDC1 support and prevents TCF1 positivity from being interpreted as completed reactivation.

The same logic applies to therapeutic remodeling. Vascular LIGHT, broad LTβR agonism, relief of TGFβ-mediated CAF restraint, and FAP-targeted LTβR agonism intervene at different levels of the niche (2, 7, 11, 27). A treatment may increase APC numbers or TCF1-positive cells without improving antigen-specific function, or may restore CD8 activity through vascular access while leaving the dominant presenting cell unresolved. Collagen alignment can alter CD8 T-cell movement in three-dimensional tissue even without TLSs, endothelium, or APCs (26). Thus, treatment-induced changes in cell number, location, and state should be reported separately from the functional output they are proposed to explain.

The most informative experiments perturb cDC1, other APCs, endothelial entry, and CAF state separately within the same model, then measure tumor-draining lymph-node output, transvascular entry, local antigen presentation, T-cell clonotypes, and tumor control in a defined temporal order. Such designs can reveal whether cDC1 are indispensable, whether another APC population compensates, or whether the principal limitation lies upstream in vascular access or stromal organization. Human cohorts can nominate the relevant spatial combinations, but they require paired tissue sampling and functional measurements before any APC or TLS feature is used to predict treatment benefit.

The anatomical route also determines where a T-cell response may be initiated or sustained. Lymphatic vessels can carry tumor-derived antigen, soluble material and antigen-bearing cells toward tumor-draining lymph nodes, where priming, clonal expansion and differentiation can occur. HEVs provide a separate blood-to-tumor entry route for circulating lymphocytes. An intratumoral TLS or perivascular aggregate may then retain, present antigen to, or restimulate cells that have entered the tumor, but its presence does not establish that priming occurred locally. These compartments can exchange cells and signals, yet they should not be collapsed into one pathway: the same treatment may increase dendritic-cell output in the draining node, lymphocyte entry through HEVs, and local T-cell activity through partially independent mechanisms. Studies that claim local reactivation should therefore distinguish node-derived expansion from intratumoral antigen presentation by combining anatomical tracing, matched node and tumor sampling, antigen-specific readouts, and cell-directed perturbation.

Human cohorts currently show mainly that this division of labor may differ among patients. TLSs are associated with better prognosis in pancreatic neuroendocrine tumors, but the relationship between TLS count and outcome is not simply linear. Some univariable associations in breast cancer disappear after adjustment, and morphological TLS burden does not consistently add prognostic information (78–80). Exceptional responses in renal cancer can arise in tumors with low TLS transcriptional signals, while an oral cancer study included only 17 baseline TLS specimens (67, 68). Small molecular subsets from a randomized mesothelioma trial and a single-arm nivolumab cohort in renal cancer can nominate biomarkers, but cannot show that TLSs or CAFs mediate treatment effects (69, 70). The absence of a significant relationship between lymphoid-aggregate burden and outcome in cervical cancer further shows that broad structural counts may miss the functional cellular combination (81). Future studies should test whether restoring a defined functional constraint improves treatment response more reliably than increasing TLS abundance alone.

## 7 Therapeutic remodeling of the CAF-vascular-immune niche

### 7.1 Therapeutic targets act at distinct mechanistic levels

Therapeutic interventions act on different constraints and should not be interpreted as a single CAF-led sequence. A local response may be limited by stromal responsiveness, vascular entry, recruitment and retention, or antigen presentation by cDC1 and other APCs. Broad LTβR agonism affects CAFs, endothelial cells, and dendritic cells at the same time; it increases HEV-associated vessels, local antigen-presentation signals, and TCF1-positive CD8 T cells while improving T-cell-directed therapy (2). The study therefore shows that several tissue functions can be remodeled together. It does not identify which cellular change is necessary for efficacy, nor does it establish that CAF remodeling precedes the vascular or APC effects.

FAP-targeted LTβR agonism concentrates the drug around FAP-positive stroma. It similarly increases HEVs and perivascular TCF1-positive CD8 T cells and improves response to checkpoint inhibition (27). Spatial targeting increases delivery specificity, but the distribution of FAP across tumor and normal tissues requires clinical evaluation. These approaches represent distinct development strategies. Broad agonism tests whether the tissue retains global responsiveness, whereas targeted delivery seeks to confine an active dose to tumor stroma. The former more readily produces systemic immune and vascular effects. The latter may be limited by target heterogeneity and drug penetration. Clinical translation should measure target occupancy, local CAF changes, and normal-tissue exposure rather than infer intratumoral action from plasma drug concentration.

Additional LTβR stimulation may be insufficient when TGFβ has fixed CAFs in a myofibroblastic state. In a pancreatic cancer model, TGFβR1/ALK5 inhibition restores CAF responsiveness to LTβR before chemotaxis, adhesion, early lymphoid aggregation, and T-cell-dependent tumor control appear (7). Ligand form also influences the response. Membrane-bound LTα1β2 combined with TNFα acts more strongly on fibroblasts than the soluble agonists tested, a finding extended by nanovesicles supplying LTα and TNFα locally (21, 49). TNFR deficiency in melanoma disrupts CAF networks and reduces checkpoint-therapy efficacy even when T-cell numbers still rise. Restoring tissue organization is not equivalent to increasing infiltration (22). Future development and comparative evaluation of LTβR- or TNFR-directed therapies should therefore account for the pre-existing CAF state, ligand-delivery mode, and any residual limitation at the vascular gateway.

### 7.2 LIGHT and innate immune therapies alter several steps simultaneously

LIGHT-based strategies illustrate a different level of intervention. Soluble LIGHT increases cytotoxic lymphocytes and cDC1-like cells, whereas vascular-targeted LIGHT creates perivascular regions containing APCs and TCF1-like CD8 T cells (11, 50). LIGHT expressed by chimeric antigen receptor T cells or delivered selectively to tumors can also increase T-cell entry, MECA-79-associated vessels, and tumor control (82, 83). Because LIGHT can signal through LTβR on stromal and endothelial cells and through HVEM on immune cells, these results support concurrent remodeling of vascular access and immune activation rather than exclusive mediation by TLS formation. Receptor-selective blockade and cell-specific deletion are required to determine whether the dominant effect is vascular, stromal, cDC1-related, or direct T-cell costimulation.

Interventions directed more directly at antigen-presenting or innate compartments provide a complementary comparison. Engineered T-bet/IL-36γ dendritic cells, local STING agonism, and an IL-15-encoding oncolytic adenovirus increase dendritic-cell activation and T-cell entry, with PNAd-associated vessels or lymphoid aggregates appearing in parallel (54, 84, 85). FLT3L-secreting cDC1 link tumor-draining lymph-node output with intratumoral antigen presentation and sustain CD8 T-cell responses despite immature TLS-like architecture (10). These findings indicate that improved APC function can compensate for incomplete stromal organization in some models. They do not show that cDC1 replace other APCs, or that the accompanying vascular and structural changes are required for the treatment effect. Blocking vascular trafficking, cDC1 function, and TLS-related signals separately is therefore necessary when the mechanism of a combination therapy is assessed.

### 7.3 The contribution of each component in engineered combinations must be resolved

Chemokine combinations and engineered materials can address several deficits at once. CCL19 with CXCL13 recruits CCR7-positive T cells and dendritic cells while positioning CXCR5-positive B cells. CXCL13 combined with CCL21 likewise increases TLS-like structures and improves anti-PD-L1 responses in melanoma (19, 25). Multi-ligand sustained-release materials prolong local chemokine and cytokine exposure, generating T-cell and B-cell aggregates and increasing antigen-reactive CD8 T cells (86). Their modularity allows cargo to be matched to a deficient step. Yet high and sustained artificial concentrations may not reproduce spontaneous TLS development. Sequence may also matter. Restoring vascular access before chemotactic recruitment can differ from establishing a cellular aggregate before stimulating antigen presentation. Sustained CXCL13 may provoke unwanted lymphoid inflammation outside the tumor. Material systems should optimize not only total release, but also release order, spatial range, and persistence after treatment withdrawal. Controls lacking each individual cargo are essential.

Engineered exosomes, TGFβ-trapping nanovesicles, and immunogenic extracellular vesicles further combine interferon signaling, myeloid remodeling, vascular improvement, and chemotaxis within one delivery system (87–89). Ectopic FRC-like cells, FRC-endothelial combinations, and stromal spheroids instead provide a reticular scaffold or engineered cellular neighborhood (3, 9, 90). These complex systems first establish that several functions can be restored concurrently, but are especially vulnerable to attributing efficacy solely to more TLSs. More discriminating designs should remove individual cargos, stagger administration, and use receptor blockade or cell depletion to resolve vascular, stromal, dendritic-cell, and direct T-cell contributions. If architecture persists after efficacy is lost, the decisive function lies downstream of the structure. If efficacy remains while TLSs decline, TLS formation is more likely an accompanying tissue change.

When local remodeling is combined with an effector therapy, the relevant initiating function must be specified. Checkpoint inhibition may benefit when LTβR agonism, chemokine delivery, vascular remodeling, or cDC1 support first improve entry, persistence, or antigen presentation; PD-1/PD-L1 blockade then acts on the available T cells (2, 10, 25, 27). Cell therapies and synthetic immune circuits can supply effectors together with organizing signals, as reported for LIGHT-expressing CAR-T cells, tumor-directed LIGHT with low-dose CAR-T cells, and an ILC3–HVEM circuit (82, 83, 91). An increase in TLSs or HEVs, or synergy between two agents, is not sufficient to identify the mediating step. Mechanistic attribution requires interruption of the proposed gateway, APC function, or organizing signal while the added therapeutic benefit is measured.

### 7.4 Safety and biomarker validation constrain clinical translation

Promoting lymphoid organization can also provoke inflammation in normal tissues. Higher plasma CXCL13 is associated with immune-related adverse events. CXCL13 neutralization reduced anti-PD-1-associated ectopic lymphoid organization and organ inflammation in aged tumor-bearing mice. In a separate young-mouse experiment, the antitumor effect of anti-PD-1 was maintained during CXCL13 blockade; tumor control remained limited in aged mice (92). These findings motivate spatially restricted treatment and careful evaluation of treatment duration, but do not establish long-term safety across cancers. FAP-targeted LTβR agonism requires assessment of normal-tissue FAP, drug exposure, antidrug antibodies, and HEV kinetics. Tolerability of tumor-directed LIGHT with low-dose CAR-T cells in mice provides only a starting point for dose escalation and off-target monitoring (27, 83). Safety evaluation should extend beyond body weight and acute cytokines. PNAd-positive vessels, lymphocyte aggregates, and organ dysfunction should be assessed in non-tumor tissues. If higher doses no longer increase intratumoral target occupancy but continue to amplify peripheral inflammation, further escalation will not widen the therapeutic window. Spatial selectivity, treatment schedule, and reversibility are as important as the maximum tolerated dose.

Patient selection cannot rely on one CAF marker, one HEV phenotype, or one TLS count. CCL19/CCL21-positive FRC-like CAFs may indicate preserved stromal responsiveness, whereas antigen-presenting CAFs, FMO2-positive CAFs, vascular markers, or cDC1-related signals may describe different local constraints (4, 6, 93). These features come largely from retrospective analyses or small treatment cohorts and lack externally validated thresholds. A clinically informative assay would need to determine, in matched tissue, whether the relevant stromal state is recoverable, whether vessels admit lymphocytes, whether cDC1 or another APC can present tumor antigen, whether reactivatable T cells remain, and whether treatment induces inflammation in normal tissue. Target presence must be separated from pathway recoverability: FAP abundance does not ensure conversion to an FRC-like state, PNAd positivity does not establish continued cellular flux, and TCF1-positive T cells do not ensure adequate nearby antigen presentation. **Table 2** organizes current interventions by the step they are intended to influence, while preserving the distinction between structural remodeling and demonstrated immune output.

**Table 2:** Major therapeutic strategies for remodeling CAFs, vasculature, and local immune responses.

Strategy class	Representative intervention	Principal step restored	Spatial and immune outcomes	Evidence stage and model	Main limitations	Representative studies
Relieve CAF restraint and restore responsiveness	TGFβR1/ALK5 inhibition; preservation of TNFR-dependent CAF networks	Inadequate CAF response to lymphotoxin and TNF	Restores chemotactic, adhesive, and reticular programs and promotes early lymphocyte aggregation	Fibroblast experiments and murine pancreatic cancer and melanoma models	State transition depends on cancer type and timing; vascular trafficking and antigen presentation are not ensured	(2, 7, 21, 22)
LTβR/TNFR agonism and lymphotoxin delivery	Broad or FAP-targeted LTβR agonism; membrane-bound or vesicular LTα/TNF delivery	Insufficient local organizing signals or non-selective delivery	Concurrent changes in CAFs, endothelial cells, and dendritic cells, with HEVs, TCF1-positive CD8 T cells, or treatment sensitization	Cell experiments and several murine tumor models	Several cell classes respond; FAP distribution and systemic inflammation require evaluation	(2, 21, 27, 49)
LIGHT-mediated remodeling of vascular gateways	Soluble LIGHT; vascular-targeted AAV-LIGHT; tumor-directed LIGHT	Insufficient endothelial activation and lymphocyte entry	Increases PNAd/MECA-79-associated vessels, lymphocyte entry, and perivascular APC-T-cell neighborhoods	Several murine solid-tumor models	LIGHT acts through both LTβR and HVEM; vascular effects and direct costimulation remain unresolved	(11, 50, 83)
Engineered LIGHT and directed delivery	LIGHT-CAR-T cells; vascular- or tumor-restricted LIGHT expression	Inadequate local LIGHT exposure and poor entry of cell therapies into solid tumors	Enhances local costimulation, T-cell infiltration, MECA-79-associated vessels, and tumor control	Engineered-cell and gene-delivery murine solid-tumor models	Direct T-cell stimulation is difficult to separate from niche remodeling; manufacturing and tissue distribution require validation	(11, 82, 83)
Chemokine combinations and sustained release	CCL19/CXCL13; CXCL13/CCL21; multi-ligand sustained-release materials	Inadequate recruitment or compartmentalization of T cells, B cells, and dendritic cells	Increases T-cell/B-cell aggregates, spatial segregation, and antigen-reactive CD8 T cells	Human spatial studies and murine intervention models	Artificial dose and timing may not recapitulate natural TLS development; sustained CXCL13 may increase inflammation	(19, 25, 52, 86)
Dendritic-cell/cDC1 expansion and innate immune activation	T-bet/IL-36γ dendritic cells; STING agonism; IL-15 oncolytic virus; FLT3L-secreting cDC1	Insufficient local antigen presentation and T-cell reactivation	Increases dendritic-cell activation and CD8 T-cell responses, sometimes with PNAd-positive vessels or immature TLS-like structures	Engineered-dendritic-cell, viral, STING, and *in situ* cDC1-vaccine murine models	Vascular, dendritic-cell, and TLS changes coincide; cDC1 necessity still requires direct testing	(10, 54, 84, 85)
Multi-cargo vesicles and local materials	Engineered exosomes; TGFβ-trapping nanovesicles; immunogenic or lymphotoxin-containing vesicles	Coexisting stromal, myeloid, and vascular abnormalities	Concurrently enhances interferon signaling, chemotaxis, vascular function, and cytotoxic T-cell responses	Nanodelivery and extracellular-vesicle murine tumor models	Component contributions, pharmacokinetics, manufacturing consistency, and normal-tissue exposure remain unclear	(49, 87–89)
FRC-like stromal and endothelial niche engineering	FRC-like cell transplantation; FRC-endothelial combinations; stromal spheroids or local scaffolds	Absence of a reticular scaffold and specialized vascular neighborhood	Generates T-cell-dominant or T-cell/B-cell aggregates, HEV-like vessels, and local antigen responses	Ectopic transplantation, peritumoral implantation, and material-engineering murine models	Most are preassembled artificial systems that remain distant from autochthonous human tumors	(3, 9, 86, 90)
Combination with checkpoint inhibition	LTβR- or chemokine-based remodeling plus anti-PD-(L)1; *in situ* cDC1 vaccination plus anti-PD-1	Checkpoint suppression of resident or newly entering T cells	Improves T-cell entry, maintenance, or effector activity and enhances tumor control in some models	Several syngeneic murine tumor models; no human mechanistic validation	Combination benefit does not establish TLS mediation, and synergy varies among models	(2, 10, 25, 27)
Combination with cell therapy and synthetic immune circuits	LIGHT-CAR-T cells; tumor-directed LIGHT plus low-dose CAR-T cells; ILC3-HVEM circuit	Insufficient entry, local persistence, and costimulation of transferred cells	Enhances cellular infiltration, HVEM-related stimulation, cytotoxicity, or immune memory	Engineered-cell therapies and receptor-ligand circuits in murine solid tumors	Direct effector activity is difficult to separate from niche effects; safety and manufacturing are complex	(82, 83, 91)

AAV, adeno-associated virus; APC, antigen-presenting cell; CAF, cancer-associated fibroblast; CAR-T, chimeric antigen receptor T cell; cDC1, conventional type 1 dendritic cell; DC, dendritic cell; FAP, fibroblast activation protein; FLT3L, Fms-like tyrosine kinase 3 ligand; FRC, fibroblastic reticular cell; HEV, high endothelial venule; HVEM, herpesvirus entry mediator; ICI, immune checkpoint inhibitor; ILC3, type 3 innate lymphoid cell; LTβR, lymphotoxin beta receptor; PNAd, peripheral node addressin; STING, stimulator of interferon genes; TCF1, T-cell factor 1; TGFβ, transforming growth factor-beta; TLS, tertiary lymphoid structure; TNF, tumor necrosis factor.

## 8 Human evidence across cancer types and its clinical boundaries

### 8.1 Spatial studies show cellular co-occurrence, not causality

Human spatial studies have identified CCL19- or CCL21-positive FRC-like CAFs in several cancers and localized them near T cells, B cells, HEVs, or TLSs (5). Their principal value is to establish that cell combinations resembling those in experimental models exist in patient tumors. They do not show that CAFs appeared first and induced the TLS. The anatomical route also matters: lymphatic drainage and tumor-draining lymph nodes can support antigen transport, priming, and clonal expansion, whereas HEVs provide a blood-to-tissue entry route and TLS-associated stroma can retain or restimulate cells after entry. Human studies rarely measure these compartments in the same patient or at matched time points. Human HEV organoids and blockade experiments in mice demonstrate PNAd-dependent trafficking, but direct migration measurements are lacking in patients (2, 9). Rescue of CAF state in pancreatic cancer and FAP-targeted LTβR delivery establish the importance of stromal state and drug localization, but cannot replace serial observations before and during treatment in patients (7, 27). Spatial findings across cancers also show that the cellular combination is not fixed. Suppressive CAFs can surround mature TLSs in lung cancer, while conventional dendritic cells and TCF1-positive T cells aggregate before complete TLS formation in liver cancer. In ovarian cancer, anatomical site and PDPN-positive stroma markedly influence the observed structure. Labeling all these configurations as “TLS-positive” discards the most informative differences. Human studies should therefore record lymphatic proximity, draining-node status when available, HEV phenotype, and local APC/T-cell states as related but non-interchangeable observations.

Different methods also measure different objects. Whole-slide histopathology is suited to TLS location and maturity. Regional imaging measures distances among B cells, CD8 T cells, and vessels, whereas single-cell and spatial transcriptomics identify CAF and endothelial states (94–96). Algorithms applied to hematoxylin and eosin images can screen lymphoid aggregates rapidly, and germinal-center scores emphasize mature structures. Neither directly measures vascular trafficking or antigen presentation (97, 98). Tissue microarrays may miss TLSs at tumor margins, while FDG-PET conflates tumor metabolism with immune organization (99, 100). Spatial barcoding can miss nearest-neighbor cells, and internal random splits of public expression data do not substitute for external validation (101, 102). Even multiplex immunofluorescence on one small biopsy may underestimate whole-tumor burden (103). An integrated strategy would first map structural location and total burden on whole slides, then sample representative tumor core, margin, and TLS regions for cell-resolved analysis. Pre-treatment and on-treatment samples should be matched anatomically whenever possible. This design reduces bias from high-resolution analysis of a very small region while preventing bulk molecular or imaging signals from obscuring local cellular relationships. Before cross-study comparison, TLS definition, sampling site, analyzed area, and primary readout should be harmonized.

### 8.2 Samples obtained before and during treatment can resolve temporal order

Pre-treatment specimens define baseline state, on-treatment specimens best capture drug action, and post-treatment resections reflect the combined effects of treatment and tumor regression. These time points should not be interpreted interchangeably. After therapy for pancreatic cancer, larger CD8 areas and more PNAd-positive vascular lumina can coexist with fewer PD-1-positive cells and more CD163-positive myeloid cells. Immune improvement and residual myeloid suppression can occur within the same tumor (104, 105). TLS features decrease after treatment in rectal cancer, whereas mature TLSs show different outcome associations after treatment in esophageal cancer. Treatment regimen and sampling time can thus change the meaning of the same marker (106, 107). In a single-arm oral cancer study, TLS density rises after therapy only among patients with a major pathological response, while baseline TLSs do not distinguish response. This finding is closer to a pharmacodynamic change than a validated predictive biomarker (108). A post-treatment decrease in TLSs does not necessarily indicate impaired immunity. Tumor shrinkage changes the denominator, existing TLSs may be replaced by resection beds or scar tissue, and immune cells may relocate to the residual tumor margin. Conversely, more aggregates after treatment may reflect inflammation without antigen-specific function. Preserving original tissue area, absolute counts, and spatial coordinates helps separate biological remodeling from changes in sampling geometry.

Appropriate comparator groups remain necessary even when post-treatment tissue is available. In a non-randomized lung cancer cohort, HEV positivity was higher after immunochemotherapy than after chemotherapy. Without paired baseline specimens, however, induction of HEVs within individual patients cannot be established (109). Studies of tongue cancer and two esophageal cancer cohorts reported either no significant change in TLS maturity or an increase in TLSs. Non-random allocation and different locations of biopsy and resection specimens can influence both findings (110–112). An early bladder cancer trial analyzed immunity in only five responders, while molecular analyses within a randomized ovarian cancer trial were limited by missing paired specimens (113, 114). Combined antiangiogenic and immunotherapy in soft-tissue sarcoma increased immune-cell infiltration and proximity without generating ordered TLSs or a clear outcome benefit (115). Paired samples improve temporal inference, but treatment mechanism requires sufficiently complete sampling, an appropriate comparator, and concurrent functional measurements.

### 8.3 Prognosis, treatment prediction, and therapeutic mechanism are distinct questions

An association between a TLS or CAF feature and survival establishes general prognostic information. A predictive biomarker for immunotherapy must additionally show an interaction between that feature and treatment. Baseline TLSs in advanced lung cancer are associated with outcomes after anti-PD-1 combination therapy, and a spatial TLS model in head and neck cancer distinguishes some responders. Both require independent cohorts and treatment comparators (103, 116). Patients with intrahepatic cholangiocarcinoma who undergo successful resection after conversion therapy are a highly selected population. A composite immune score can describe outcomes in that population, but should not be generalized directly to all patients (117). The clinical comparison in a randomized ovarian cancer trial is stronger than its small molecular subset, and post-treatment HEV findings are not consistently significant across lung cancer cohorts (109, 114).

A predictive study should prespecify a biomarker threshold within a design containing treatment and control groups, then test whether relative treatment benefit differs by biomarker state. If patients with high TLS burden survive longer regardless of therapy, TLSs are more likely prognostic. A treatment-selection role is supported only when relative benefit differs between TLS-high and TLS-low groups. The model must also improve decisions after established clinical variables are included, rather than report statistical significance alone. Prognostic association, baseline prediction, and post-treatment tissue change should be analyzed separately.

Establishing treatment mechanism requires a stricter temporal and causal sequence. Treatment should first alter CAFs, HEVs, or antigen presentation, followed by changes in T-cell function and clinical outcome. Interruption of the intermediate step should then weaken the effect. LTβR agonism, TGFβR1 inhibition, and FAP-targeted delivery provide components of this evidence in preclinical models, but human studies have not connected them along one within-patient timeline (2, 7, 27). Window-of-opportunity trials should record dose, intratumoral target engagement, normal-tissue exposure, and anatomically matched pre-treatment and on-treatment biopsies. CXCL13-associated systemic inflammation should be monitored concurrently (92). An early bladder cancer trial can inform safety and tissue change, but its size is insufficient to assess rare toxicity or establish TLS-mediated efficacy (113). **Table 3** summarizes human evidence by cancer type, separating spatial architecture, potential immune function, treatment association, and the conclusions currently supported.

**Table 3:** Human evidence for CAF-, HEV-, and TLS-associated immune responses across cancer types.

Cancer type or evidence group	Principal spatial pattern	Relationship among CAFs, vessels, and immune cells	Functional or treatment association	Conclusion currently supported	Representative studies
Non-small cell lung cancer	TLS neighborhoods can contain FRC-like CAFs, CXCL13-positive tumor-reactive CD8 T cells, and distinct HEV states	Some mature TLSs contain suppressive FAP/αSMA-associated CAFs; ID1-high HEVs, CD4-helper-Tpex neighborhoods, and B-cell-plasma-cell compartmentalization indicate more active local responses	Baseline TLS composition, endothelial state, and post-treatment spatial modules associate with response or survival, inconsistently across cohorts	TLS presence alone is insufficient for stratification; CAF state, HEVs, and T-cell function should be assessed together	(5, 28, 59, 60, 72, 101, 109, 116, 118, 119)
Head and neck cancers, including nasopharyngeal cancer	CCL19/CCL21-positive FRC-like CAFs occupy B-cell and CD4 T-cell zones of TLSs; some lesions contain PNAd-positive or CXCL9-positive MECA-79-positive vessels	Lymphotoxin responsiveness accompanies chemokine expression, while interferon-responsive HEVs accompany lymphocyte aggregation; structural completeness differs by anatomical site	Baseline spatial features or post-treatment TLS increases associate with response in small cohorts; prognostic effects of maturity are inconsistent	Supports stromal-vascular-lymphocyte co-occurrence; treatment-predictive thresholds require prospective validation	(4, 8, 67, 75, 76, 103, 108, 110)
Pancreatic ductal adenocarcinoma	Reticular CAFs localize closer to TLSs than TGFβ-associated myofibroblastic CAFs; post-treatment tissue can contain PNAd-positive vessels near CD8 T cells and residual myeloid suppression	Pre-existing CAF state may constrain lymphotoxin responsiveness; neural, metabolic, and therapeutic influences jointly alter local organization	The prognostic direction of TLS burden varies by context; post-neoadjuvant changes are primarily pharmacodynamic	The strongest causal evidence is experimental; human studies support state differences and post-treatment remodeling	(7, 36, 37, 100, 104, 105)
Colorectal cancer and liver metastases	FRC-like CAFs, IL-36γ-positive vessels, and PNAd-positive HEVs contribute to graded TLS networks; CCL19/CCL21-CCR7 and CXCL13 jointly organize T cells and B cells	CAFs recruit CCR7-positive cells and chemokine combinations promote compartmentalization; TLS-associated IgG in liver metastases can recognize tumor antigens	TLS location, germinal centers, and post-treatment change have different clinical meanings; transcriptional scores may capture general inflammation	Human evidence supports spatial organization and some humoral function, but no standardized quantification	(19, 20, 33, 40, 55, 98, 106, 120)
Esophageal squamous cell carcinoma	Intratumoral, peritumoral, and distant mature TLSs have distinct distributions; TLS-rich regions often contain more plasma cells and less FAP	Tfh-B-cell, germinal-center, and plasma-cell programs indicate humoral activity; post-treatment structural changes are inconsistent	Location and maturity provide prognostic information in untreated cohorts; TLS increases associate with response or disease-free survival in some neoadjuvant cohorts	Spatial location is more informative than simple counts; baseline treatment prediction remains unvalidated	(39, 107, 111, 112)
Gastric cancer	MHC-II/CD74-high antigen-presenting CAFs are enriched in TLS regions; a CXCL13-CXCR5-positive CD8 axis coexists with TLSs	Antigen-presenting CAFs can support CD4 T-cell activation and indirectly enhance CD8 responses; TGFβ-associated regulation occurs within the same structures	Tissue antigen-presenting-CAF or CXCL13 features associate with outcomes in small anti-PD-1 cohorts; serum CXCL13 stratification is inconsistent	Supports participation of antigen-presenting CAFs in local immunity, but not a validated treatment biomarker	(6, 41, 121)
Hepatobiliary cancers	CCL21-positive stroma, activated conventional dendritic cells, and TCF1-positive memory-like CD8 T cells can precede complete TLSs in hepatocellular carcinoma; MECA-79-positive HEVs coexist with TLSs in cholangiocarcinoma	FMO2-positive CAF-CCL19 signaling, CCR7 recruitment, antigen presentation, and HEV influx may provide complementary functions	Spatial class, CAF/CCL19 features, and HEVs separately associate with treatment outcome or survival in liver cancer; mature TLSs do not consistently predict conversion-therapy outcome in cholangiocarcinoma	HCC and iCCA show related directions but distinct biology; both lack standardized thresholds and human causal validation	(61, 62, 93, 117, 122)
Melanoma	CD8/CD20 aggregates adjoin HEV-like vessels and lymphatics; nerves, LTA-positive memory B cells, and stromal B-cell aggregates alter the local state	Vascular activation, the memory B-cell reservoir, and TCF1-positive T-cell neighborhoods jointly reflect recruitment and maintenance	TLS number and quantified B-cell aggregates separately associate with survival or checkpoint-therapy outcome	Quantified spatial features outperform simple presence or absence; predictive value requires independent validation	(25, 44, 51, 94, 96, 123)
Clear-cell renal cell carcinoma	CXCL13-positive CD8 T cells, TLSs, regulatory T cells, and tumor-associated macrophages can coexist; spatial neighborhoods differ from bulk transcriptional signatures	CD8 expansion can coincide with higher checkpoint expression and reduced effector function; organization does not ensure function	CXCL13-positive CD8 T cells associate with poor prognosis in untreated cohorts; immunotherapy cohorts show candidate or non-significant associations	Represents active architecture with impaired function; TLSs should not be used alone for treatment stratification	(68, 70, 77, 95)
Ovarian cancer	The cellular source of CXCL13 changes with TLS stage; normal mesenchymal-like stroma is more TLS-supportive, whereas PDPN-positive CAF-rich regions can show plasma-cell skewing and incomplete maturation	Tumor FAK, CAF state, anatomical site, and CXCL13/CXCR5 jointly influence B-cell and T-cell organization	Prognostic effects of TLSs depend on site and stroma; tissue subsets from a randomized neoadjuvant trial provide exploratory associations only	TLSs cannot be interpreted without anatomical site and CAF state; treatment mediation is unproven	(24, 29, 31, 43, 73, 114)
Breast cancer	MECA-79-positive HEVs colocalize with lymphocyte-rich TLSs; TLS proportion and location vary among molecular subtypes	HEVs indicate a potential vascular gateway, but evidence for antigen responses within TLSs and CAF contributions is limited	Survival associations in whole cohorts and triple-negative breast cancer are mostly null or significant only before adjustment	Subtype, definition, and sampling differ substantially; current evidence mainly supports structural description	(58, 63, 79, 80, 124)
Soft-tissue sarcoma	TLS-like regions can contain perivascular macrophages; tissue microarrays can miss structures, and greater proximity after treatment does not produce ordered TLSs	Myeloid context may constrain structural maturation; infiltration and proximity do not establish function	Combined antiangiogenic and immunotherapy does not induce mature TLSs or show clear TLS-associated benefit	Demonstrates how sampling and structural definitions can determine conclusions	(42, 99, 115)
Bladder cancer	IGLL5-positive B cells associate with abnormal HEVs and absent TLSs; responding specimens can contain FRCs, follicular dendritic cells, and segregated T-cell/B-cell zones	B-cell state, vasculature, and stromal chemotaxis jointly influence organization	The abnormal-HEV axis associates with immunotherapy non-response; neoadjuvant and phase I tissue analyses are small	Supports HEV dysfunction and treatment-associated remodeling, but not a predictive biomarker	(32, 64, 113)
Other cancers with limited evidence	Merkel cell carcinoma shows shared T-cell-receptor clones between TLSs and tumors; mesothelioma shows TLSs with inflammatory infiltrates; penile cancer contains CCL21-positive CAFs near CCR7-positive B cells	Individual cancers suggest clonal continuity, T-cell/B-cell cooperation, or CAF-associated recruitment, but not one shared mechanism	Treatment associations in Merkel cell carcinoma and mesothelioma arise from small tissue subsets; penile cancer and other tumors rely mainly on retrospective spatial associations	Supports cancer-specific hypotheses, not pooled efficacy estimates or cross-cancer causal extrapolation	(35, 69, 74, 78, 81)

apCAF, antigen-presenting cancer-associated fibroblast; CAF, cancer-associated fibroblast; cDC, conventional dendritic cell; DFS, disease-free survival; FAP, fibroblast activation protein; FDC, follicular dendritic cell; FRC, fibroblastic reticular cell; HEV, high endothelial venule; HNSCC, head and neck squamous cell carcinoma; ICI, immune checkpoint inhibitor; IFN, interferon; LTβR, lymphotoxin beta receptor; NSCLC, non-small cell lung cancer; PNAd, peripheral node addressin; TAM, tumor-associated macrophage; TCF1, T-cell factor 1; TCR, T-cell receptor; Tfh, T follicular helper cell; TGFβ, transforming growth factor-beta; TLS, tertiary lymphoid structure; TNBC, triple-negative breast cancer; Tpex, progenitor exhausted T cell. The qualifier “-like” retains the approximate phenotype or model definition used in the source study.

The human evidence therefore supports a narrower conclusion than the preclinical experiments. Patient studies can show where CAF, HEV, APC and TLS features coexist, how those features relate to prognosis, or how they change during treatment. They have not yet demonstrated in one patient cohort that a treatment first changes CAF state, then increases HEV-mediated entry, then produces APC-dependent local T-cell reactivation that mediates tumor control. These observations should be reported as distinct clinical evidence settings rather than combined into a validated treatment mechanism or a response-prediction rule.

## 9 Discussion

### 9.1 TLS abundance and local immune function frequently diverge

TLS abundance, local immune function, and benefit from immunotherapy often change independently. In some tumors, outcome does not improve simply as TLS counts rise. Associations in univariable analyses can disappear after adjustment, while marked responses to checkpoint inhibition can occur in patients with low TLS transcriptional signals (68, 78, 79). Conversely, more immune cells and shorter intercellular distances after treatment do not necessarily produce ordered TLSs. Mature TLSs can also coexist with FAP- or αSMA-associated CAFs, impaired T-cell function, and resistance to immunotherapy (115, 119). These observations are not merely conflicting study results. TLS morphology records how far cells have been organized, but not where they entered, whether they encountered antigen, or whether they can kill tumor cells. Anatomical location further changes the meaning of the same structure. TLSs in the tumor core, invasive margin, and adjacent tissue encounter different antigens, vessels, and suppressive populations. One count on a two-dimensional section can also represent three-dimensional structures with very different volumes and connectivity. Timing adds another distinction. Baseline TLSs reflect the pre-existing immune environment, on-treatment changes are closer to drug action, and post-treatment resections combine regression with changes in sampled area. Without location, denominator, and timing, an “increase” or “decrease” in TLSs does not carry a consistent meaning across cohorts.

Structure and function must be observed separately. A morphologically developed TLS accompanied by vascular trafficking, antigen presentation, and T-cell effector activity is more likely to participate in local immunity. When structure is developed but function is weak, HEV trafficking, suppressive CAF matrix, and dendritic-cell sufficiency should be examined. An incomplete structure with demonstrated T-cell entry and antigen responses may be a functional immature niche, or may reflect an alternative route provided by endothelial cells or cDC1. When both structure and function are low, sampling that missed the tumor margin remains a possibility. The same assessment should be repeated before and during treatment because preserved architecture does not imply unchanged cellular composition, location, or function. This distinction does not require a new TLS grading system. It shows why one structural measure cannot represent every immune process.

### 9.2 Organizing functions are distributed and context dependent

The division of labor summarized here is a cross-model comparison of experimentally separable functions, not a sequence demonstrated within one tumor system. Evidence for stromal recruitment, blood-to-tumor entry, and local antigen-dependent responses comes from different cancers, anatomical compartments, and perturbation designs. We therefore retain the model, tissue location, intervention, and measured endpoint for each study and use the synthesis to identify relationships that should be tested together. A complete CAF–HEV–APC cascade should not be inferred unless one system demonstrates the temporal order, component-specific perturbations, functional readouts, and tumor consequence within the same experiment.

CAFs are neither a fixed nor a unique starting point across cancers. The available evidence instead supports a division of labor. CAFs recruit and support immune cells; endothelial cells control entry from blood; cDC1 and other APCs restimulate T cells; and B cells, CD4 T cells, macrophages, and innate lymphoid cells provide maintenance or amplification. CAF importance cannot be inferred from TLS proximity. CAF-specific perturbation should alter recruitment, trafficking, or local activation, and endothelial or cDC1 enhancement should be tested for rescue. Cross-perturbation within one model can then distinguish an indispensable CAF, a supportive cell whose function is compensable, and a change that merely accompanies inflammation.

This division of labor also changes the question for translational research. Broad LTβR agonism, relief of the TGFβ-associated myofibroblastic state, and FAP-targeted LTβR delivery address insufficient signaling, CAF unresponsiveness, and poor drug localization, respectively (2, 7, 27). TLSs in patient tumors may increase, decrease, or accompany opposing immune changes after treatment, indicating that therapy often restores only part of the process (106, 107). Small tissue subsets from randomized trials and single-arm cohorts primarily nominate biomarkers and cannot independently demonstrate TLS-mediated benefit (69, 70). CXCL13-associated inflammation in normal tissue also requires systemic safety assessment when intratumoral organization is enhanced (92). Future studies should determine whether CAF responsiveness, a vascular gateway, and local antigen presentation can be measured as distinct functional constraints and whether their restoration predicts treatment activity.

The relative amount of evidence for each component also reflects the available assays rather than a fixed biological hierarchy. CAF state may remain stable while endothelial entry limits the number of relevant lymphocytes reaching the tumor, and adequate entry may coexist with insufficient cDC1 or other APC function. Independent HEV and APC analyses are therefore required even when a stromal association is present. This separation keeps the review centered on experimentally testable functions and prevents the larger CAF literature from being read as evidence that CAFs are the universal upstream determinant.

**Figure 2** separates the spatial description of a niche from the experimental tests of its functions. Panel A is a composite tissue view showing B- and T-cell zones, reticular stroma, a PNAd-positive vascular gateway, tumor nests, and the spatial proximity of cDC1 and a TCF1-positive CD8 T cell. The adjacent reticular aggregate illustrates an incompletely organized follicular configuration; vascular LIGHT, FLT3L-secreting cDC1, and immune-cell-derived CXCL13 are shown as parallel organizer inputs rather than as a sequential pathway. Panel B presents three independent assays: transferred-cell recruitment with PNAd blockade tests lymphocyte entry; peptide-loaded and washed cDC1 co-cultured with OT-I CD8 T cells tests antigen-dependent T-cell proliferation; and anti-CD8 depletion during tumor treatment tests the contribution of CD8 T cells to tumor control. Arrows in panel B denote operations within each assay and do not connect the three experiments. The figure integrates findings from separate studies and models; it is not a reconstruction of one tumor or a universal CAF–HEV–APC cascade. TCF1 positivity identifies a cell state and does not by itself establish antigen-dependent reactivation. OT-I cells identify the antigen-specific cells used in the coculture assay and are not labeled as intratumoral TCF1-positive progenitors. Cell numbers, relative sizes, and tissue geometry are schematic.

**Figure 2:**
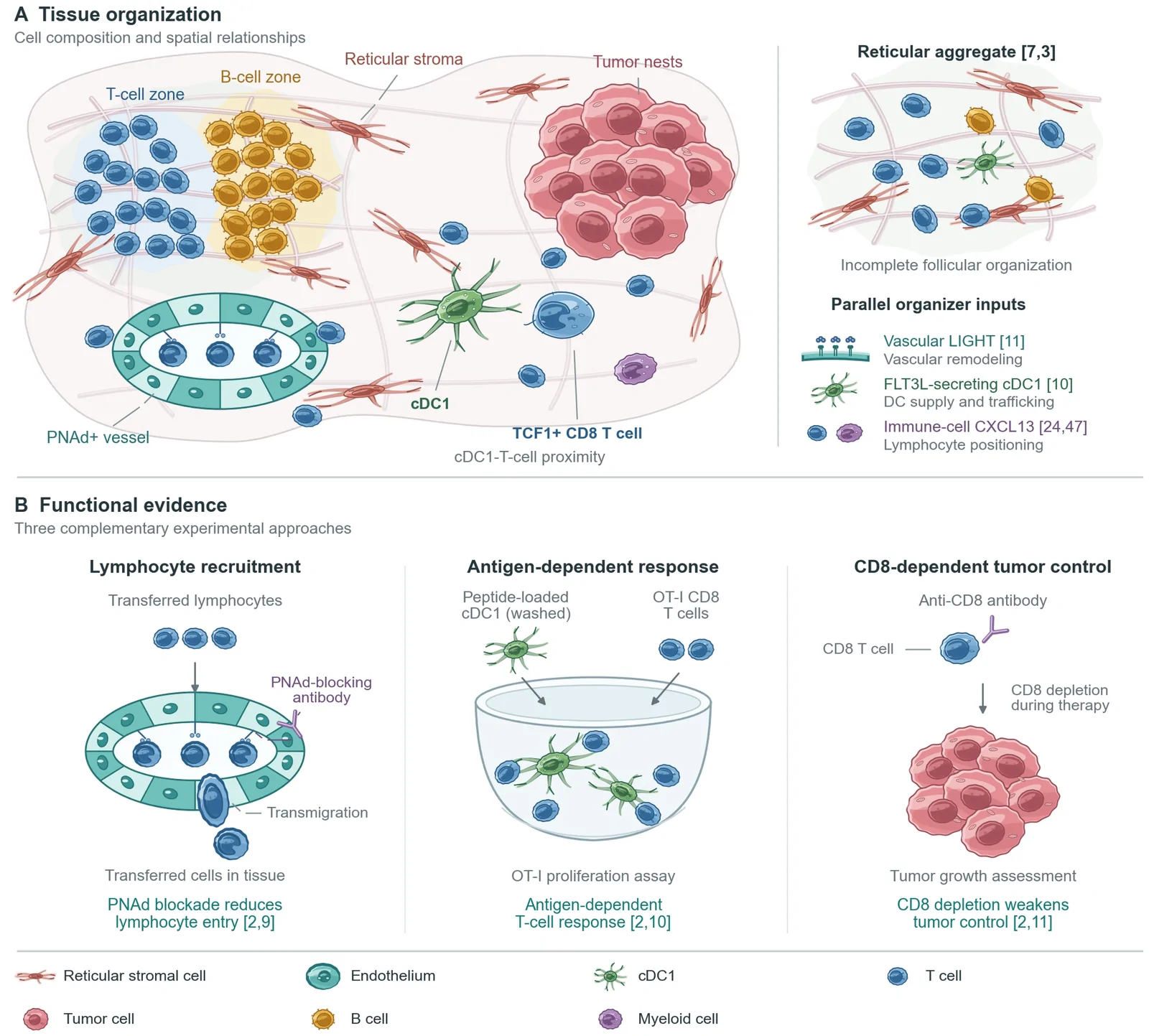
Tissue organization and functional assessment of TLS-associated immune niches. **(A)** Composite view of B/T-cell zonation, reticular stroma, PNAd-positive vessels, tumor nests, and cDC1-adjacent TCF1-positive CD8 T cells. The reticular aggregate illustrates incomplete follicular organization in pancreatic CAF (7) and engineered lymph-node-derived stromal-cell (3) models. Parallel organizer inputs include vascular LIGHT and vascular remodeling (11), delivery and trafficking of FLT3L-secreting cDC1 (10), and immune-cell-derived CXCL13 in lymphocyte positioning (24, 47). **(B)** Three independent experimental approaches examine distinct immune functions. Left, transferred-cell recruitment with PNAd blockade tests lymphocyte entry in tumor and engineered HEV-transplantation models (2, 9). The elongated lymphocyte is crossing the vascular wall; the antibody beside the vessel represents the PNAd-blocking reagent. Middle, antigen-dependent DC-T-cell assays assess T-cell responses (2, 10). The illustrated coculture follows reference 10: cDC1 are peptide-loaded and washed before addition of OT-I CD8 T cells, whose proliferation is then measured. Right, CD8 depletion during tumor therapy tests the contribution of CD8 T cells to treatment-associated tumor control (2, 11). The panels synthesize separate studies; arrows within B denote assay procedures. Cell numbers, relative sizes, and tissue geometry are schematic. PNAd-bearing addressins are shown at the luminal endothelial surface. TCF1 identifies a T-cell state in A, whereas OT-I identifies the antigen specificity of the cells used in the coculture assay in B. CAF, cancer-associated fibroblast; cDC1, conventional type 1 dendritic cell; DC, dendritic cell; FLT3L, FMS-like tyrosine kinase 3 ligand; HEV, high endothelial venule; LIGHT, TNFSF14; OT-I, ovalbumin-specific T-cell-receptor-transgenic CD8 T cells; PNAd, peripheral node addressin; TCF1, T-cell factor 1; TLS, tertiary lymphoid structure.

### 9.3 Current evidence gaps

The first gap is inconsistency in the object being measured. TLS count, maturity, spatial score, and transcriptional signature represent structural abundance, follicular development, cellular position, and mixed expression signals, respectively. They cannot be converted into a single common threshold. When an original study reports a “lymphocyte aggregate”, “TLS-like structure”, or “candidate HEV”, a subsequent review should retain that qualification rather than rename the feature as a mature TLS. TCF1, PD-1, and proliferation markers may indicate that CD8 T cells have the potential for maintenance or reactivation. Confirmation of Tpex identity and local reactivation still requires antigen specificity, restricted recirculation, clonal tracking, or cellular perturbation.

The second gap is the disconnect between causal and human evidence. CAF-specific state changes, blockade of HEV-mediated trafficking, and cDC1 perturbations arise mainly from murine or engineered models. Patient studies are well suited to describe spatial states, longitudinal changes, and clinical outcomes. Few show within one study that a CAF change precedes improved T-cell function, and fewer demonstrate that interrupting a CAF-associated process diminishes treatment activity. A further practical gap is intratumoral spatial heterogeneity: CAF state, vascular phenotype, antigen-presentation capacity, and T-cell clonotypes can differ between the tumor core, invasive margin, necrotic regions, and TLS-adjacent areas. A single biopsy may therefore capture one local configuration while missing another region that contributes more strongly to treatment response. Differences in cancer type, sampling time, and analytical scale create additional apparent inconsistencies. Cross-study comparisons can nominate mechanisms only when tumor context, intervention timing, sampling location, and functional readouts are comparable. Spatially matched sampling of more than one tumor region, with sampled area and coordinates retained, is needed before a local feature is generalized to the whole tumor. Fragments from different models should not be assembled into a pathway that appears fully established.

### 9.4 A path for mechanistic validation and clinical studies

This Review does not propose a universal marker panel, numerical score, or functional threshold for patient stratification. The cited studies use different CAF, endothelial, and APC definitions, and a threshold derived in one tumor or assay cannot be transferred without independent validation. A minimum within-study operational package can nevertheless be stated: the candidate cell state should be defined before perturbation; its direct output should be measured in the relevant compartment; and a component-specific perturbation should be linked to that output and to the downstream tumor or T-cell endpoint. For CAFs this means pairing state markers with chemokine, adhesion, or matrix readouts; for HEVs, an endothelial phenotype with directional blood-to-tissue entry; and for APCs, cell identity with antigen-specific presentation or T-cell function. These are measurement requirements, not a validated composite score.

The decision rule should remain experimental and context-specific. A component is more plausibly necessary when its targeted perturbation reduces the direct function and the downstream endpoint, whereas preserved efficacy after that perturbation suggests compensation or a nonessential contribution. Rescue, orthogonal perturbation, or cross-component comparison can distinguish these possibilities. Studies should sample baseline and on-treatment tissue, and when feasible matched tumor-draining nodes, with post-treatment tissue interpreted separately because regression changes the sampled denominator. In patients, a TLS, CAF, or HEV feature should be considered a candidate pharmacodynamic or stratification marker only after its treatment interaction and functional correlate are tested in a comparative cohort; this Review does not supply a clinical decision threshold.

Early clinical trials can follow the same sequence. Intratumoral drug exposure and target engagement should be established first. On-treatment biopsies can then measure CAF state and HEV changes, followed by local antigen-specific T-cell responses and clinical outcome. If CAFs or vessels change as intended but T-cell function does not improve, insufficient antigen presentation or absent effector cells should be investigated instead of escalating the same agonist. CAFs, HEVs, or TLSs can support treatment selection only after biomarker-treatment interactions are demonstrated in comparative studies. Systemic CXCL13-associated adverse events and selective tissue sampling in early trials also require concurrent assessment of normal-tissue inflammation and specimen representativeness (92, 113). Until then, these features are better regarded as candidate stratification markers and tissue pharmacodynamic readouts.

## 10 Conclusion

In the models reviewed, defined FRC-like CAF states can translate lymphotoxin, TNF, and other inflammatory signals into CCL19, CCL21, adhesion molecules, and reticular matrix. These outputs help recruit and organize T cells, B cells, and dendritic cells. Not all CAFs share this function. A TGFβ-associated myofibroblastic state reduces responsiveness, while neighboring suppressive myeloid cells, tumor cells, and tissue mechanics modify the final outcome. CAFs also cannot complete the sequence alone. Endothelial cells govern entry into the tumor, and local persistence and reactivation require cDC1, other APCs, and additional immune-cell signals.

A mature TLS can support effective immunity, but it is not the only functional architecture. Less complete structures may permit T-cell entry and activation, whereas morphologically mature TLSs can remain deficient because of poor vascular trafficking, absent antigen presentation, or suppressive CAFs. The framework yields testable predictions. Within one model, selective restoration of CAF responsiveness should increase chemotaxis and retention without necessarily increasing antigen-specific killing if vascular entry or APC function remains limiting. Blocking HEV-mediated entry should reduce the accumulation of newly arriving clonotypes even when TLS morphology is preserved. cDC1 depletion or antigen-MHC interruption should selectively reduce local T-cell-receptor signaling and effector output if APC-dependent restimulation is the limiting step. In patients, matched samples from tumor core, margin, TLS region, draining-node context, and on-treatment tissue should reveal whether these functional changes occur in the predicted order. These measurements, rather than a single CAF marker, HEV phenotype, or TLS score, should determine whether a combination strategy has restored the step that was limiting.
